# Ensuring Continuity of Transitional Housing for Homeless Veterans: Promoting Disaster Resilience among the Veterans Health Administration’s Grant and Per Diem Providers

**DOI:** 10.1177/2150132719861262

**Published:** 2019-07-17

**Authors:** June L. Gin, Roger J. Casey, Jeffery L. Quarles, Aram Dobalian

**Affiliations:** 1 Veterans Emergency Management Evaluation Center (VEMEC), U.S. Department of Veterans Affairs, North Hills, CA, USA; 2 VA National Center on Homelessness Among Veterans, U.S. Department of Veterans Affairs, Tampa, FL, USA; 3 National VA Grant and Per Diem Program, U.S. Department of Veterans Affairs, Tampa, FL, USA; 4 School of Public Health, University of Memphis, Memphis, TN, USA

**Keywords:** access to care, qualitative methods, homelessness, disasters, preparedness, emergency preparedness, vulnerable populations, community health

## Abstract

The US Department of Veterans Affairs (VA) has committed significant resources toward eliminating homelessness among veterans as part of its health care mission. The VA Grant and Per Diem (GPD) program funds non-VA, community-based organizations to provide transitional housing and support services to veterans experiencing homelessness. During a disaster, GPD grantee organizations will be especially critical in ensuring the well-being of veterans residing in their programs. Recognizing the need to ensure continued access to this residential care, the VA GPD program implemented a disaster preparedness plan requirement for its grantee organizations in 2013. This study conducted semistructured interviews with leaders of 5 GPD grantee organizations, exploring their perceptions of the preparedness requirement, the assistance they would need to achieve desired preparedness outcomes, and their motivations toward preparedness. Organizations reported being extremely motivated toward improving their disaster preparedness, albeit often for reasons other than the new preparedness requirement, such as disaster risk or partnerships with local government. Two dominant themes in organizations’ identified needs were (1) the need to make preparedness seem as “easy and doable” as possible and (2) the desire to be more thoroughly integrated with partners. These themes suggest the need to develop materials specifically tailored to facilitate preparedness within the GPD nonprofit grantees, an effort currently being led by the VA’s Veterans Emergency Management Evaluation Center (VEMEC).

## Introduction

People experiencing homelessness face significant health risks from being unhoused. Exposure to the elements, the lack of a stable nightly place to sleep, and other daily challenges of homelessness make it very challenging, if not impossible, to address basic health care needs and manage chronic illnesses such as hypertension, asthma, and diabetes. For people experiencing homelessness, acute infections and injuries may be prolonged and aggravated by not having a place for recovery. Moreover, the risk of contracting communicable diseases such as hepatitis and tuberculosis is elevated. Serious mental health and substance use disorders are also difficult to treat while staying in temporary shelters or being unhoused.^1-4^

Lack of access to housing and shelter negatively impacts access and health outcomes.^3,5^ Patients without housing are more likely to be readmitted to hospitals and tend to stay much longer than housed individuals, resulting in higher costs to the health care system.^1,3^ Increasing awareness of the link between housing and health has resulted in growing recognition of the importance of residential housing programs, including transitional housing, residential treatment, vouchers for permanent supportive housing, and other housing programs as a vital component of health care for people experiencing homelessness.^1,2^

In 2009, the US Department of Veterans Affairs (VA) embarked on an effort to eliminate homelessness among veterans, increasing resources and services to facilitate their efforts to transition into stable permanent housing. In 2010, veterans represented 16% of all chronically homeless adults and 13% of the sheltered homeless population while comprising only 9.5% of the total adult population. As federal agencies and their partners have dedicated resources to providing housing and services, homelessness among veterans has declined by 48.4% since 2009.^6^ Despite these gains, veterans still face greater risk of experiencing homelessness than other adults due to several risk factors, including challenges in reintegration to civilian life, health concerns, and trauma.^7-9^

### VA Homeless Programs and VA Grant and Per Diem

The VA’s focus on funding community nonprofit organizations to provide housing to homeless veterans as part of its health care mission began in 1992 with the Congressional authorization of the VA Grant Per Diem (GPD) program. Since 1994, the VA GPD program has annually funded non-VA, community-based organizations to provide transitional housing and other services to veterans experiencing homelessness. Veterans can stay for up to 2 years with the goal of helping them achieve residential stability, receive treatment, increase their skills and income, and achieve community reintegration.^9-12^ Annually, 23 000 veterans are served in the GPD program, with an average of 11 000 veterans receiving GPD services on any given day. Grantees assume responsibility for the veteran’s housing, welfare, and progress toward identified goals.^13^ GPD grantee organizations can be found in every geographic region across the United States, including Puerto Rico and the US Virgin Islands. They vary significantly in size, with some organizations having only 5 to 10 GPD dedicated beds and others with as many as 100 to 160 GPD dedicated beds within a larger organization housing hundreds of residents. Some GPD programs serve male veterans exclusively, while others have programs for both genders. Some common GPD service models are (1) bridge housing for veterans in the process of transitioning into an identified permanent housing placement, (2) clinical treatment programs for veterans with substance use or mental health disorders, and (3) service-intensive transitional housing.

GPD grantees are inspected annually by an interdisciplinary team from the associated VA Medical Center (VAMC), led by a social worker called a “GPD liaison,” who monitors the veterans’ progress. Each GPD liaison works with an average of roughly 2 organizations, often monitoring several service programs within each organization. The GPD inspection covers a wide scope of areas, including life safety, security, staffing, licensure, sanitation, clinical care, and services for veterans.

### Disaster Vulnerability, Nonprofit Organizations, and the GPD Disaster Plan Requirement

During a disaster, GPD grantee organizations will be even more critical in ensuring the continued well-being of the veterans residing in their programs, who often have limited to no alternative options for housing. These organizations are themselves vulnerable to closure or disruption after disasters when their services may be most needed. Nonprofit homeless service providers are often inadequately prepared for disasters, and lack continuity of operations plans to enable them to quickly resume operations after a disruption.^14-17^ Community-based organizations (CBOs) often face difficulty in locating guidance for their disaster preparedness efforts, and typically lack incentives to engage in preparedness planning due to limited budgets and lack of technical expertise and guidance. These organizations are often not required by their funding agencies to have disaster response plans beyond basic life safety codes.^18^

If these organizations were to lose their ability to provide these essential daily support services after a disaster, it would substantially disrupt the continuity of care that their clients need to recover from the trauma of homelessness. Concerns about disaster preparedness among GPD grantees became a focal point for the VA GPD program in the aftermath of Hurricane Katrina in 2005, which affected several GPD grantees and resulted in resident evacuations. In 2013, VA GPD leaders adopted a new disaster plan regulation requiring all GPD grantees to have a written disaster plan that is coordinated with emergency managers for their local jurisdiction. This new requirement is included in the GPD annual inspection.^19^

This disaster plan requirement was not accompanied by any written guidance or technical assistance for grantees. The VA GPD program does not require its GPD liaisons to take any standard training in organizational disaster preparedness; thus, they may not have the technical expertise to assist organizations in writing plans. VA GPD leaders have observed tremendous variation in the degree to which GPD facilities have improved their disaster preparedness planning in response to this new requirement. This lack of consistency in disaster preparedness among nonprofit human service organizations has been documented in previous research.^15,16^ Most funding entities generally do not attach disaster preparedness requirements to their funding, although some larger funders require grantees to have written disaster plans.^20^

To better understand how GPD grantees have navigated this new requirement, this study examined 5 GPD grantee organizations and their perceptions of the disaster preparedness task—how feasible it would be for them to achieve objectives and what they needed to attain desired outcomes. The study team sought to understand factors that motivated and assisted GPD organizations’ preparedness efforts, and their needs to assist with preparedness.

## Methods

This was an exploratory study of preparedness in nonprofit organizations participating in the VA GPD program. Participants were identified through convenience sampling. They were recruited by the VA National Center on Homelessness Among Veterans, who identified VA GPD grantee organizations and contacted them to invite them to participate in the study of disaster preparedness within GPD grantee organizations. Five GPD grantee organizations were contacted, and all 5 organizations agreed to participate in the study. These organizations were in the eastern United States and ranged in size from 10 to 89 veterans served in their GPD programs. Three of the 5 organizations consisted exclusively of programs for male veterans, while the other 2 were multiservice organizations serving a range of clients of both genders, including veterans in the GPD program. According to policies regarding activities that constitute research at the VA Greater Los Angeles Healthcare System, this study met criteria for quality improvement activities and was exempt from human subjects review.

In 2015, one member of the senior leadership staff was interviewed at each of the 5 GPD organizations. During these interviews, organization staff were asked to describe (1) forms of assistance they received in their preparedness planning efforts, (2) disaster plan requirements they were required to meet, and (3) training, technical assistance, or other resources they would need from funders or government agencies to improve their preparedness capabilities. Interviews were digitally recorded, and researchers took detailed notes from the recordings to compile transcripts from the interviews. Researchers returned to the recordings to verify any discrepancies.

Transcripts were iteratively analyzed using Atlas.ti (version 7.5.17, Berlin, Germany) qualitative analysis software. Interviews were coded using both an inductive *grounded theory* approach of identifying themes that from the data that were most salient to the organizations’ perspectives and within 3 areas of deductive domains governing the interview guide.^21^ *Thematic analysis* was conducted using both theoretical and inductive approaches, focusing on identifying factors that contributed to the motivation for preparing for disasters.^22^ The first author read through the transcripts and identified broad thematic categories that were salient to understanding the organizations’ perspectives. The authors were particularly interested in identifying subthemes within the broad categories of sources of motivation and needs for preparedness. These broad categories were central to the research question of what factors motivated organizations to prepare, and what they needed to assist them with preparedness. These codes were grouped by shared content. The significance of themes was based on their *substantive significance*,^23^ referring to the extent and context in which these themes were present in the data. Transcripts from interviews were examined using a constant comparative method^24^ to confirm that the themes identified were consistent across the narratives.

## Results

GPD grantees find it difficult to promote preparedness within their organizations. Some GPD leaders noted receiving assistance from government partners but indicated that complying with disaster planning requirements is often frustrating because they are not offered help or funding for preparedness. However, they identified training and technical assistance areas where they believed that targeted guidance, information, and resources, along with collaboration with peer organizations and the VA, could improve preparedness.

### Sources of Motivation

GPD grantees found it difficult to commit to preparedness within their organizations due to limited time, resources, and lack of buy-in from organizational management. However, several factors motivated organizations to prepare, including prior experiences with hurricanes, free resources and training, and engagement and assistance from partners. Their perceptions of the effectiveness of disaster plan requirements were mixed. One respondent, located in a major city in the northeastern United States, noted that most organizations tend to only do “what’s minimally required”:

Funding always helps. You’re better off bringing resources if you’re going to have a mandate, you need to make the process doable… Non-profits just don’t have the “person power” to dedicate to planning.

A second organization leader, in the same city, also expressed mixed views about whether requirements helped motivate preparedness given their resource constraints:

I would not say requirements create enthusiasm. Just because there’s a requirement, that doesn’t make it easy for an organization to meet them. Agencies that administer the (grants) should think about how they can provide needed help and support, so grantees can execute the requirements and keep the motivation and traction to get it done.

More optimistically, this leader noted that while buy-in is a challenge, they successfully generated internal momentum for preparedness with the help of the local public health department providing free assistance, and testimonials from other service providers who experienced Hurricane Sandy in New York.

The other three organizations were all located in the Gulf Coast and noted that their geographic location—in areas frequently affected by hurricanes, motivated them to prepare, irrespective of any requirements. As one organization director put it:

We are motivated because of our geographic location. Florida takes disasters seriously, because we’re highly populated and had some years where we had 5 hurricanes. We also have fires in the area…

This organization cited the VA GPD’s long-standing life safety requirements as a factor in contributing to their proactive disaster planning for two decades. This organization viewed the 2013 VA GPD’s disaster plan requirement as simply a formal reinforcement of its requirement to have life safety protection measures in place, as its director noted,

The disaster plan was required as part of the VA GPD program, so it was established by staff about 20-25 years ago. . . . VA GPD asks about the disaster plan and asks to review it every year when we have an inspection. It has always been required as part of our GPD grant.

They took an expansive view the GPD life safety requirement, viewing it as an impetus to engage in disaster preparedness planning. As an affiliate of a national nonprofit organization, the site’s disaster plan was also mandated by their parent organization, which provided a template for local sites to adapt to their needs. This organization also noted that assistance from the VA GPD liaison also motivated their preparedness.

### Outside Assistance

Outside partners played a critical role in motivating preparedness actions. Organizations noted that in the absence of preparedness funding, free resources, including training, technical assistance, and supplies were helpful to motivate organizations internally. One organization had the local public health department conduct disaster preparedness training as part of a citywide initiative to recruit nonprofit agencies to provide community outreach during disasters:

So that was the reason for buy-in—free training, free resources for individuals and organizations. Non-profit social service agencies have very limited resources, so the prospect of getting free resources is always a way to get buy-in.

Other organizations noted that their county emergency managers ensured that homeless service providers were included in county emergency plans:

Emergency management officials come into our organization…. They continually come out to the agency due to our temporary population.

### Needs—Training and Technical Assistance

GPD grantee organizations cited numerous areas where training and technical assistance were needed. They needed assistance to build buy-in for preparedness and mentioned that standardized templates to guide them through the process of creating their own disaster plans and training their staff would be helpful. An outline of best practices, tailored to the needs and constraints of homeless service organizations would be ideal, they said, covering who to call in disasters, where to go, and how to deal with transportation and meals.

Organizations with more thorough disaster plans agreed that other homeless service providers that did not have the resources available to them would likely face difficulties in creating a disaster plan from the beginning. As one commented,

If organizations don’t have a disaster plan, they need someone to help them create a plan. Especially mom and pop GPD organizations, they really need assistance since they don’t have the resources to do it on their own.

Multiple respondents cited the need for guidance on collaborating with community partners to communicate and have a coordinated disaster plan. They also stressed the importance of guidance and assistance building networks to communicate with funders, emergency managers, and federal partners such as HUD and the VA during disasters.

### Needs—Ideal Format for Training

Respondents were also asked about their preferred format for training and assistance. All respondents stressed the importance of training that is accessible to all staff and having an “internal champion”—someone leading the preparedness effort within each organization, noting that having external partners follow up regularly was crucial to sustaining momentum. Congregate phone calls, webinars, or local meetings would enable organizations to learn from each other and share challenges and solutions. However, there was some divergence in respondents’ preference for web-based training versus in-person congregate training. Respondents tended to favor training with more interactive opportunities:

Congregate training is best since it allows us to bounce things off other people, and to hear others’ ideas and insights. Being physically present helps a lot to keep one focused without distractions, as opposed to a webinar.

### Needs—What GPD Organizations Want From the VA

GPD grantees stressed that additional support and resources were needed from the VA and other funders to help achieve preparedness. Organizations also emphasized the need to make a case for sustaining motivation for preparedness among staff, through training that seems “easy and doable,” and something compelling to articulate why preparedness matters, that is, one’s own disaster experiences or those of others.

One organization director expressed a desire to be more thoroughly integrated with their VAMC emergency management plans:

We drive veterans to the VA every day to appointments, but we don’t have a relationship with the VA for emergency management coordination. It would be good to have that stronger relationship with the VA.

A protocol for GPDs to work with the VA in a disaster would be a step toward addressing this concern, he noted. While GPD grantees typically assume responsibility for the safety and well-being of veterans enrolled in their programs, extenuating circumstances, such as the evacuation of veterans during Hurricane Katrina, have required the VA to assume responsibility for sheltering veterans in the GPD program.

One organization, located in the Gulf Coast, had extensive plans outlining evacuation protocols, as its director describes,

We have to make sure we know where veterans are going and that each staff member has a location to go to and how we will communicate with staff… daily during a disaster. We have to make sure we stay in communication if we relocate elsewhere.

Organizations that benefitted from active involvement by the VA in their disaster planning expressed appreciation for this support, which likely contributed to their sense of being well integrated with their local VAMC’s disaster plans.

## Discussion

GPD grantee organizations face difficulties in disaster preparedness planning due to resource and personnel constraints and the lack of technical guidance and assistance to orient them through the process. This finding is consistent with previous studies,^15-17^ that have found such barriers to preparedness prevalent in homeless service organizations. Typically, organizations perceived preparedness requirements as unfunded mandates. They were skeptical that such mandates alone could serve as an impetus to drive motivation for preparedness within organizations like theirs.

However, many of the organizations’ directors that were interviewed identified other sources to motivate these crucial preparedness activities. Given their expressed need for technical guidance and training, the availability of outside partners to offer free staff training, walk them through the planning process, and provide free supplies was a tremendous boost to buy-in. Conditions that made them aware of disaster risk, such as their location in a hurricane zone, also helped build internal momentum. One organization mentioned that longstanding GPD life safety requirements led them to create their plans 20 years ago. This suggests that some organizations may take a more expansive view of basic life safety requirements.

Assistance from outside partners was cited as a major factor in helping organizations gain internal momentum for preparedness, confirming prior research^16^ that established external technical assistance and training as factors in motivating preparedness. Unlike past work where outside training was identified only as a wish list item, this study found organizations that had benefitted from actual training provided by local government partners. While not all organizations will be fortunate enough to have access to local government partners who can come to their facility, non-profit organizations may benefit from forming relationships with local government, which could lead to new opportunities for funding and training. It also suggests that at least some grantees of the GPD, which sends VA employees to non-profit organizations, would be receptive to preparedness training.

Two dominant themes in organizations’ identified needs were (1) the need to make preparedness seem as “easy and doable” as possible and (2) the desire to be more thoroughly integrated with partners. These themes echo previous research, which found that an impetus to motivate preparedness, outside assistance, and collaboration with outside partners are key areas of need to motivate preparedness in homeless service providers.^16^ When organizations caution that preparedness training must seem “easy and doable,” they are warning that if recommended actions seem too challenging, motivation will erode. Entities seeking to motivate preparedness must reduce perceived barriers and increase perceived rewards.

Divergent responses to queries about preferred training formats suggest that there is no “one size fits all” solution for delivering training. Instead, offering a diverse array of options to nonprofit staff would likely be ideal. Finally, organizations emphasized the need for protocols and a clarification of roles with the VA as to how they would collaborate to ensure the safety of veterans in an evacuation situation.

### Limitations and Future Implications

These findings are likely to be transferable to building our understanding of what motivates nonprofit homeless service organizations to prepare. However, that transferability may be limited by factors that make this sample of organizations unique. These are likely exemplary organizations by virtue of their status as VA-funded partners located in hurricane-prone states and are thus likely to be particularly motivated to commit to preparedness. The study did not include any organizations in the western or midwestern United States, which would be prone to different natural hazards. Organizations facing earthquakes or wildfires might have different perceptions of disaster risk and operate on a different calculus as to what would motivate their preparedness commitment.

Secondarily, the organizational respondents might have been more motivated toward preparedness than an “average” nonprofit organization. Organizations were selected to participate in the study because of their familiarity to VA staff and because they tended to be active in national-level VA activities. In addition, organizations that are more active in such activities may have greater staff capacity and thus greater ability to participate in preparedness programs.

Finally, nonprofit organizations participating in the VA GPD grant funding program are selected from a competitive process, based on their ability to demonstrate effectiveness, and must pass a rigorous initial inspection, including over 100 regulatory criteria, to receive their grants. Organizations selected for funding through this competitive grant process are likely to be better resourced and have achieved industry best practices, possibly including better life-safety plans, as compared to organizations that have not received such grants. Therefore, the preparedness capabilities reflected in these interviews may not be reflective of nonprofit homeless service organizations in general.

Future research examining how other types of government-funded nonprofit organizations build disaster resilience through preparedness would be valuable to our understanding of the field. The GPD program is unique in its funding of nonprofits on a per bed basis, enabling the VA to conduct a detailed annual inspection of the facility and ask tough questions about preparedness planning. Examining organizations that receive grants requiring less oversight than the GPD program would enable researchers to better understand how preparedness could be fostered on a more voluntary basis with less direct intervention from funders. Given that other nonprofit funding arrangements operate with significantly less direct oversight, examining preparedness in such organizations might yield insights, for example, about potential common concerns in disaster plans, that can be generalized to a larger population of government-funded nonprofit organizations.

## Conclusion

The VA’s investment in GPD transitional housing for veterans experiencing homelessness constitutes a vital part of veterans’ health care. To prevent disruption to these services during disasters, the VA GPD program has implemented a disaster plan requirement aimed at motivating preparedness and encouraging grantee organizations to consider how they would respond in a disaster. These interviews suggest that without additional sources of motivation or assistance, grantees will continue experiencing challenges to their efforts to generate momentum for preparedness. Without such supports, preparedness adoption is likely to be uneven, with organizations in regions that frequently experience hurricanes and similar recurring events with the most robust disaster planning, while other organizations often achieve only the bare minimum needed to meet the requirement.

Outside assistance, including training and resources, and the opportunity to develop collaborative relationships with community partners was a significant source of motivation for organizational preparedness, echoing past research.^16^ This study suggests that these outside inputs create a sense of motivation by effectively reducing perceived barriers and increasing perceived rewards. Offering a diverse variety of training formats, making training and instructional materials about disaster plans accessible to a wide audience of readers, and ensuring that the process seems “easy and doable” are additional avenues for reducing perceived barriers.

Because providing individualized outside assistance to all GPD organizations is unfeasible, efforts are currently underway to create materials tailored specifically to the needs of non-profit homeless service providers, to walk organizations through the steps of disaster planning. The VA GPD program is collaborating with researchers at the Veterans Emergency Management Evaluation Center (VEMEC) to identify and develop training materials for GPD grantees. These efforts build on an interagency toolkit cataloguing best practices in disaster preparedness for homeless service providers,^25^ developed in collaboration with partners from the US Department of Health and Human Services and US Department of Housing and Urban Development. Creating instructional resources specifically developed to fit the needs of homeless service providers, these efforts represent a substantially needed investment in disaster resilience for veterans experiencing homelessness.
